# An Unusual Case of Congenital Hepatic Fibrosis with Retinitis Pigmentosa

**DOI:** 10.7759/cureus.5788

**Published:** 2019-09-27

**Authors:** Nazish Butt, Ali Akbar, Anoshia Fahad

**Affiliations:** 1 Gastroenterology, Jinnah Postgraduate Medical Centre, Karachi, PAK; 2 Internal Medicine, Jinnah Postgraduate Medical Center, Karachi, PAK

**Keywords:** congenital hepatic fibrosis, retinitis pigmentosa

## Abstract

Congenital hepatic fibrosis (CHF) is a rare hereditary autosomal recessive disorder due to periportal fibrosis and ductal plate malformation. It is just one of many different malformations collectively named oculo-encephalo-hepato-renal syndrome. The major presenting feature is upper gastrointestinal bleeding due to portal hypertension secondary to the development of esophageal varices. Herein we report a case of CHF with retinitis pigmentosa but lacking the distinctive multisystem malformations associated with other well-known syndromes associated with CHF. In cases of CHF, search for other organ involvement is important at the time of presentation as well as during follow-up.

## Introduction

Congenital hepatic fibrosis (CHF) is a rare autosomal recessive hereditary liver diseases characterized by periportal fibrosis and abnormal proliferation of bile ducts [1]. The onset of signs and symptoms varies widely ranging from childhood to the fifth or sixth decade of life but frequently occurs in adolescent and early adulthood [2]. The most common clinical manifestations result from portal hypertension and include splenomegaly, varices, and upper gastrointestinal bleeding [3]. It is a part of various clinical syndromes and associated with cystic diseases of the kidney and liver [4]. Herein we present a case of CHF with retinal lesions that does not fit with the well-known syndromes associated with CHF.

## Case presentation

**Figure 1:**
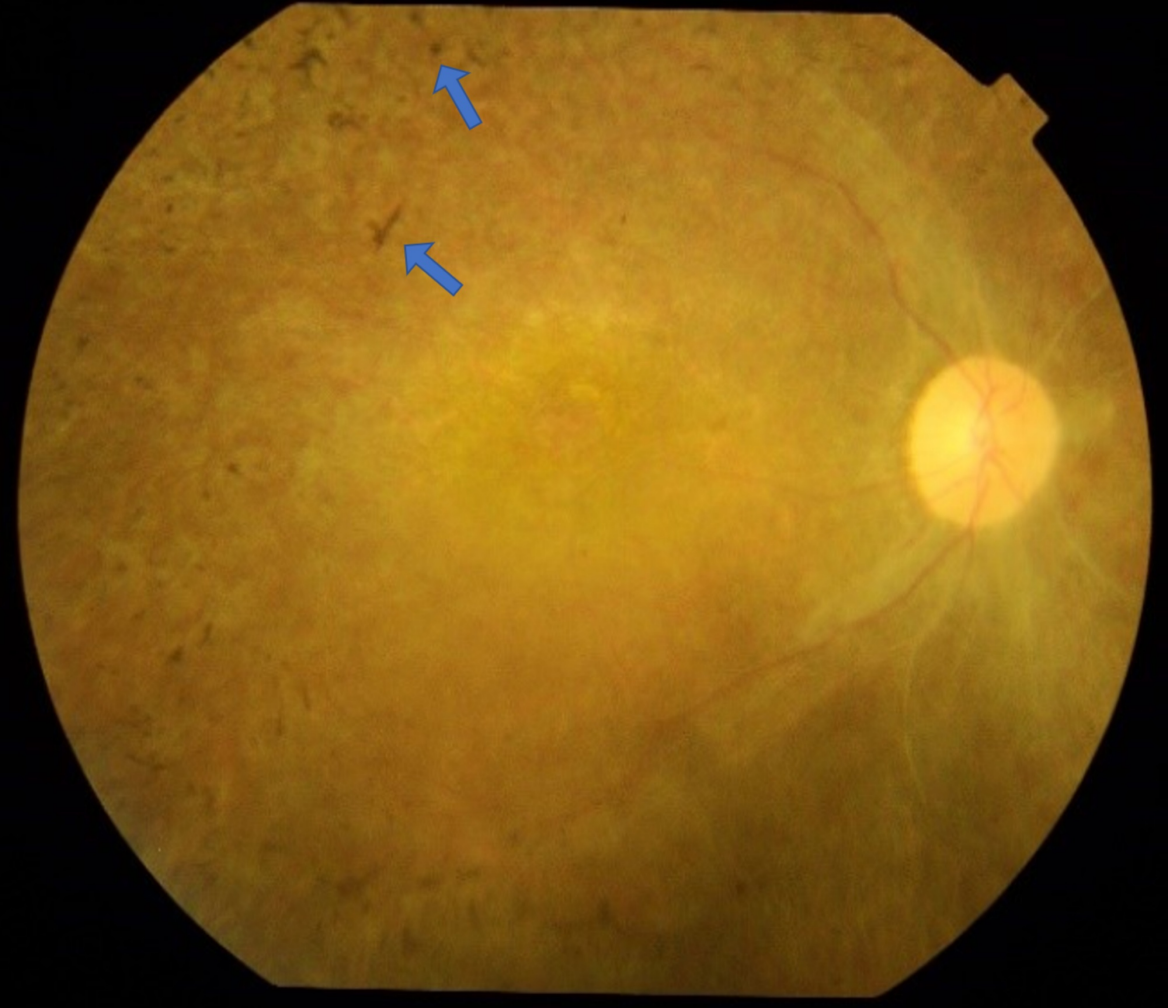
Pigment deposition in the retina.

**Figure 2:**
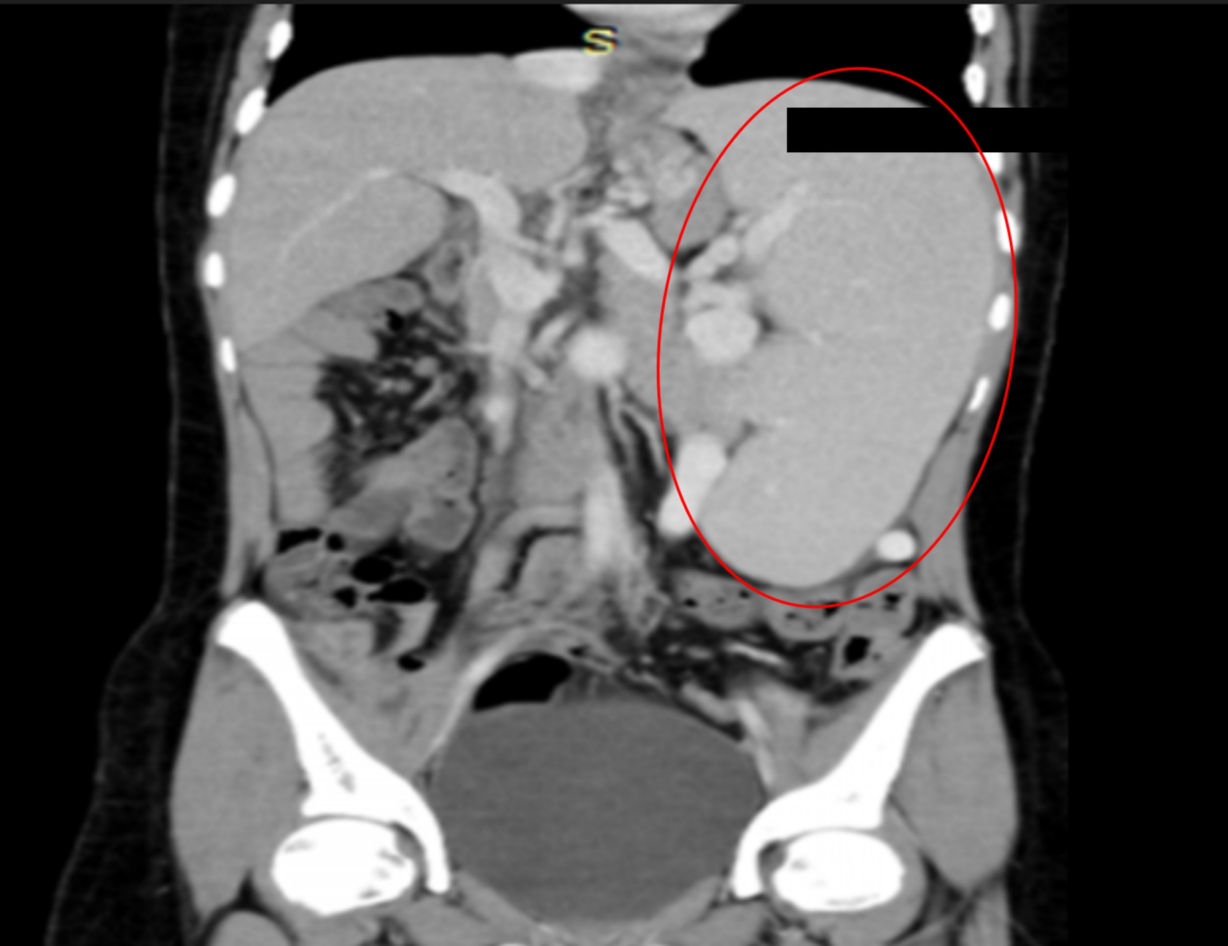
CT scan showing a dilated portal vein with splenomegaly and splenic varices.

A 17-year-old boy, third by birth order, with five siblings and born of a first-degree consanguineous marriage presented with three episodes of hematemesis and the insidious onset of progressive left abdominal discomfort. He was blind since childhood. There was no history of abdominal distension, jaundice, altered level of consciousness, or seizures. His elder sister also had blindness and renal problems and died at the age of nine years; two other sisters died at the ages of three and nine months, respectively, from unknown causes. His weight, height, and body mass index were 29 kg, 132 cm, and 16.6 kg/m^2^ (below the 25th percentile), respectively. On examination, he was pale and had massive splenomegaly. There were no Kayser-Fleischer rings found on eye examination. During the investigation, the patient’s hemoglobin was 6.4 g/dl, total leucocyte count was 3.3 × 10^3^/μl, and platelet count was 64 × 10^3^/μl. Liver function tests revealed his total bilirubin was 1.68 mg/dl, aspartate aminotransferase was 56 IU/L, alanine aminotransferase was 28 IU/L, and his international normalized ratio was 1.13 sec. The patient sample was not reactive for hepatitis B surface antigen or anti-hepatitis C virus antibodies. His detailed urine report was normal. His autoimmune and metabolic workups were also found to be normal. An upper gastrointestinal endoscopy of the patient revealed large esophageal varices with red wale marks and severe portal hypertensive gastropathy. A fundoscopy found a pale optic disc and featureless retina with bony spicules; these features are consistent with typical retinitis pigmentosa (Figure 1). Doppler ultrasound of his abdomen revealed hepatosplenomegaly with features of portal hypertension. These findings were further confirmed via computerized tomography scan that revealed massive splenomegaly of about 22 cm and hepatomegaly (Figure 2).

**Figure 3:**
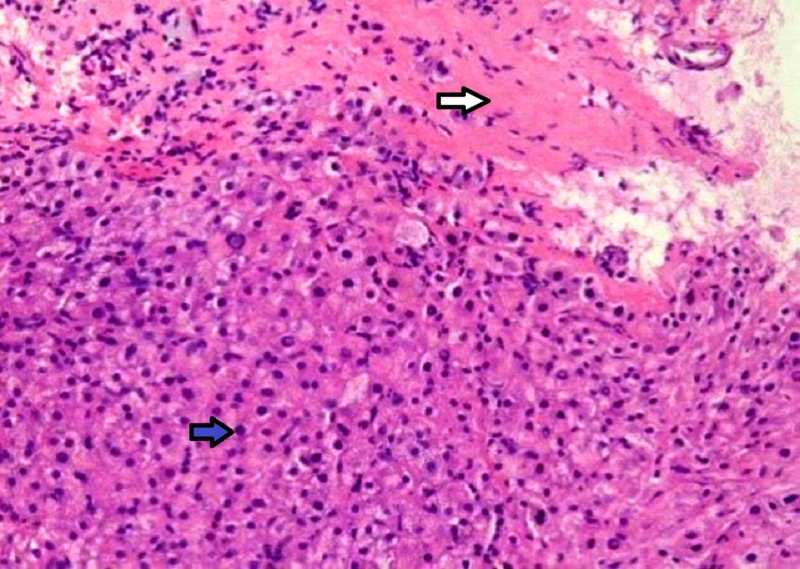
Liver biopsy showing altered architecture with lymphocytes, plasma cells (blue arrow) and bands of fibrosis surrounding the portal tracts (white arrow).

A liver biopsy found the architecture of the portal tracts altered and containing lymphocytes, plasma cells, eosinophils, and proliferating bile ducts with bands of fibrosis surrounding the portal tracts suggestive of congenital hepatic fibrosis (Figure 3). The patient was recommended for regular follow-up and referred to the liver transplant unit.

## Discussion

CHF is one of the fibrocystic diseases along with polycystic kidney disease, Caroli disease, and choledochal cyst [4]. Affected individuals are called ciliopaths because of mutations in ciliary proteins [5]. During embryonic development, the ductal plates begin remodeling around the portal veins and form biliary ductules. The lack of this phenomenon leads to the development of fibrosis around the portal vein and its branches resulting in portal hypertension [6].

CHF is often found in combination with renal, cerebellar, and other abnormalities [7]. It is a part of various clinical syndromes that includes COACH [8], Meckel [7], Arima [9], Joubert [10], and Bardet-Biedl syndrome (BBS) [1]. Joubert syndrome is distinguishable by congenital hepatic fibrosis, atypical retinitis pigmentosa, hypotonia, ataxia, nystagmus, and nephronophthisis [11], whereas BBS is a combination of CHF, atypical retinitis pigmentosa, postaxial polydactyly, central obesity, mental retardation, hypogonadism, and renal dysfunction [12]. CHF and retinitis pigmentosa are common in both syndromes along with the involvement of various other organs.

In a study from northern India, the prevalence of congenital hepatic fibrosis in children with portal hypertension was found to be 3% and the most common clinical presentation was upper gastrointestinal bleeding [13]. In our case, upper gastrointestinal bleeding was the presenting feature.

Ciliopathies are an evolving group of disorders with a wide spectrum of different presentations. In our case, the absence of major anatomical defects in the kidney, gonads, and hands (polydactyly) found in BBS and a lack of brain anomalies present in Joubert, COACH, Arima, and Meckel syndromes, point towards the possibility of a new entity with less drastic complications in comparison to other well-known syndromes. CHF and retinitis pigmentosa are found in both Joubert and BBS syndromes along with the involvement of other organs. As no other organ involvement was found in our case, this new syndrome may lie somewhere between Joubert and BBS syndrome.

In terms of treatment, no modality has been found to either stop the pathogenesis of the disease or reverse it. However, medical and endoscopic management of varices and intercurrent cholangitis is done if these develop. If there are refractory bleeding and advanced disease, a shunt procedure and transplant can be considered. These patients require screening for hepatocellular carcinoma and screening for additional system involvement during the future course of the disease.

## Conclusions

Ciliopathies are evolving disorders affecting multiple systems. The clinical manifestation depends on the penetrance of associated mutations. This patient presented with congenital hepatic fibrosis and retinitis pigmentosa. As pure CHF is rare, searching for the involvement of additional organs is important at the time of presentation as well as during follow-up.
